# The effect of the mental health first-aid training course offered employees in Denmark: study protocol for a randomized waitlist-controlled superiority trial mixed with a qualitative study

**DOI:** 10.1186/s12888-015-0466-1

**Published:** 2015-04-14

**Authors:** Kamilla B Jensen, Britt R Morthorst, Per B Vendsborg, Carsten R Hjorthøj, Merete Nordentoft

**Affiliations:** Copenhagen University Hospital, Research Unit, Mental Health Centre, Copenhagen, Kildegårdsvej 28, entrance 15, 4th floor, DK 2900 Copenhagen, Denmark; Danish Mental Health Foundation, Hejrevej 43, DK 2400 Copenhagen, Denmark

**Keywords:** Mental health, Randomized trial, Focus group, MHFA, Educational training program, Mixed-methods design

## Abstract

**Background:**

Studies show a high and growing prevalence of mental disorders in the population worldwide. 25% of the general population in Europe will during their lifetime experience symptoms related to a mental disorder. The Mental Health First Aid concept (MHFA) was founded in 2000 in Australia by Kitchener and Jorm, in order to provide the population with mental health first aid skills. The aim of the concept is, through an educational intervention (course), to increase confidence in how to help people suffering from mental health problems. Further, secondary aims are to increase the mental health literacy of the public by increasing knowledge, reduce stigma and initiate more supportive actions leading towards professional care. An investigation of the effect of MHFA offered a Danish population is needed.

**Methods:**

The design is a randomized waitlist-controlled superiority trial, in which 500 participants will be allocated to either the intervention group or the control group. The control group will attend the course six months later, hence waiting list design. From fall 2013 to spring 2014 participants will be educated to be “mental health first-aiders” following a manualized, two days MHFA course. All the participants will answer a questionnaire at base-line and at 6 months follow-up. The questionnaire is a back-translation of the questionnaire used in Australian trials. The trial will be complemented by a qualitative study, in which focus groups will be carried out.

**Discussion:**

Outcomes measured are sensitive to interpretation, hence a challenge to uniform. This trial will add to the use of a mixed-methods design and exemplify how it can strengthen the analysis and take up the challenge of a sensitive outcome.

**Trial registration:**

https://clinicaltrials.gov identifier NCT02334020.

## Background

The World Health Organization estimates a high and growing prevalence of mental disorders worldwide [1]. In Europe, a lifetime expectancy of mental health problems in the general population is estimated to be 25% [2]. Recent research from Denmark shows that the figures might even be higher – as many as 38% of the female population and 32% of the male population is expected to receive treatment in the secondary mental health sector [3].

The psycho-social and economic impact of mental disorders is considerable [4]. Early intervention can lower both the human and economic costs [4,5]. Even though the numbers might be high, we know that according to international epidemiological investigations, the rates of help-seeking behavior for mental health problems are generally low compared to how many people that actually suffers from mental health problems [6]. This is a problem because early help-seeking can enhance the opportunity for early intervention, and can therefore improve long-term outcomes.

There is evidence to suggest the effectiveness of community-based interventions in order to increase the help-seeking behavior of people with suicidal behavior [7]. The key element of such interventions is enhancing the knowledge of mental disorders through educational training of the general practitioners, municipal officials, and the general public [8]. Mental Health First Aid (MHFA) is based on such a concept.

MHFA was developed by Betty Kitchener and Anthony Jorm in Australia in 2000. The overarching aim of MHFA is to increase the mental health literacy of the general public. An increase in mental health literacy means an increased confidence in how to help people suffering from mental health problems by providing relevant knowledge, reducing stigma associated with mental illness, and providing guidance regarding supportive actions [9].

In Denmark, a report on stigma and mental illness concluded that the majority of the population in general has contact with people suffering from mental illness through e.g. their social network or workplace. At the same time, the report and others alike stated that most people suffering from mental illness have experienced discrimination [10-12]. Despite the reported contact, knowledge of mental illness is low in the general public, while there on the same time is a great interest in the public to gain more knowledge on the subject [10].

Although research to date has shown promising results of the MHFA, further replication is needed in order to ascertain the generalizability of the findings also to Danish social and cultural circumstances. Evaluations of MHFA from both Swedish and Australian trials show increased helping behavior, greater confidence in providing help to others, improved attitudes and decreased need for social distance to people with mental illnesses [13,14]. Furthermore, a qualitative study has reported that positive effects were experienced both intra-personally (through increased empathy, understanding, and confidence to act appropriately) as well as inter-personally (through increased capacity to handle crises, manage strained relationships, and offer help in an effective way) [15].

### Objective

The objective of the Danish evaluation is to investigate the effect of MHFA as an education-based intervention on increased confidence in help-giving behavior as primary outcome by comparing an intervention group and a waiting list group. Secondary outcome will be comparing increased knowledge of and improved positive attitudes towards people suffering from a mental health problem also between the intervention and the waiting list group.

Through qualitative data we wish to explore the process of becoming a mental health first-aider and the participants’ experience of the course.

### Hypothesis

We expect to find; a significant difference in confidence in helping a person suffering from mental illness between the intervention group and control group; a significant increase in knowledge of mental illness; and hopefully a significant improvement in attitudes and decrease in wish for social distance towards people suffering from mental illness [9,13,14].

## Methods/Design

The design of the study will combine concurrent and sequential elements within a mixed-methods convergent parallel design [16]. This choice of design is based on the nature of the objective of the research being both exploratory and confirmatory at the same time [17]. In the study, qualitative and quantitative data will be collected.

The design of the quantitative part is a randomized, waitlist-controlled superiority trial, in which 500 participants will be allocated to either the intervention group or the control group. The control group will attend the course 6 months later, hence waiting list design. From fall 2013 to spring 2014 participants will be educated “First Aid-helpers” following a manualized, two days MHFA course. All the participants will answer a questionnaire at baseline and at six months follow-up. Concurrently, the trial will be complemented by a qualitative study, in which five focus groups will be held with participants before and after they attend the course and participant observation will be carried out on the course.

### Recruitment to the quantitative survey – criteria for inclusion

The MHFA training program was initially developed for the general public [13]. However, international experiences show that participants most often have been people who in their line of work are in contact with many different individuals [14]. We will try to replicate that in this study.

Hence, the study will recruit employees of 10 different workplaces, including public, private and non-governmental organizations (NGOs) sectors. Possible workplaces could be social services and job centers in different catchment areas of Denmark and telephone helplines in NGOs. A characteristic of the sample would then be its geographical diversity but with some occupational homogeneity.

The recruitment will be managed by an independent assistant at The Danish Mental Health Foundation. Contact will be established to leading officials at the different workplaces and NGOs, with whom the organization of participation at the given workplace will be arranged according to firm logistics. Participation will be presented as an opportunity for an education-based training course, relevant to the participants’ work life as well as private life. Participation will be voluntary and the training course will be held during work hours. Informed consent will be required and held in qualitative parts of the study.

### Randomization and blinding

The randomization will be computer generated, provided by an independent research assistant. Allocated outcome will be informed to the leading contact at each workplace and then passed on to each participant. The randomization procedure will ensure adequate sequence generation and allocation concealment. Furthermore, the analysis of the quantitative data will be blinded. It will not be known to the researcher, which group was the intervention group or the control group before the analysis is done.

### The intervention – the MHFA training course

MHFA is a manualized, educational preventive strategy structured in a 12-hour course delivered over two days of training. The course will be carried out and managed by The Danish Mental Health Foundation.

MHFA is based on four training sessions of three hours each. The course gives an overview of the major categories of mental health disorders and crisis. The items presented include depression, anxiety, psychosis, and substance abuse. The crisis situations covered are; thoughts of suicide and suicidal behavior, self-harming behavior, panic attacks, reactions to trauma, acute psychosis, substance intoxication, and aggressive behavior. Participants are introduced to symptoms of the illness, possible risk factors, evidence-based treatment and where and how to get help [18]. The training will consist of a mix between knowledge-presentations and exercises, and a five-step action plan will be applied to each category of the mental health problems presented.

The action plan consists of a series of five steps, which include 1) Access risk of suicide or harm 2) Listen non-judgementally 3) Give reassurance and information 4) Encourage the person to get appropriate professional help, and 5) Encourage self-help strategies.

Further, the training involves cases and encourages the participants to bring in examples from their own experiences. All instructors are certified teachers and all experienced in working with people who have experienced mental health illness.

### Survey procedure

A link to the electronic questionnaires will be sent out at baseline and at six-month follow-up by email to the participants, primarily to their work email-address, in order to avoid loss to follow-up [14]. Reminders to complete questionnaires will be provided at both baseline and at follow-up.

The software program used for the distribution of the questionnaires will be Survey Xact [19].

### Instruments in the quantitative survey

The questionnaire is a back-translation of the questionnaire used in Australian trials [18]. It is translated into Danish and retranslated into English, thus validated in relation to language difference.

The questionnaire will start with a range of socio-demographic questions, motivation for participation, personal mental health history, and previous experience with persons suffering from mental illness, and what kind of help, if any, participants had offered in these situations.

Following, the primary outcome will be measured through three questions. The questions will address how confident the person feels 1) to contact a person with mental health problems 2) to talk to a person with mental health problems 3) to help a person with mental health problems. All three questions will be answered on a four step scale: “not confident at all”, “a little confident”, “quite confident”, and “very confident”. Furthermore, if the person answers “not confident at all”, he/she will have to answer another question on the primary cause for this within a five step scale: “lack of knowledge”, “afraid of saying something wrong”, “afraid of the person’s reaction”, “don’t know what to advice the person to do”, “it is too great a responsibility”.

The following questions will address the secondary outcome on increased knowledge, improved attitudes and decreased need for social distance. For this, the questionnaire contains vignettes on depression or schizophrenia as a descriptive part of suggested intervention and response options. According to possible intervening suggestions the answers will be given on a three categories scale: “helping”, “make it worse”, “no effect”.

The questionnaire will include two scales assessing social distance towards people suffering from a mental illness. The same vignettes as before mentioned will be used in the attitude assessment, which will have to be answered within a five categories scale: “fully agree”, “partly agree”, “neither agree, nor disagree”, “partly disagree”, “fully disagree”. Assessments will address the participant’s point of view for both him/her-self and for the public in general. In the first scale the respondent will be asked to state, “to which extent yourself…” and in the second scale “what you think other people think”.

The last part of the questionnaire will consist of knowledge-questions concerning how to act around people suffering from a mental illness and which treatment strategies that are most appropriate. The questions will have to be answered within the categories: “Agree”, “disagree”, “Don’t know”.

### Focus groups

Focus groups will be conducted before and after participants attend the course. The selection of participants to the focus groups will be non-strategic and criteria for participation will be expressed interest in discussion themes related to mental illness and completion of baseline questionnaire. Randomization allocation outcome will still be unknown to investigating researcher.

We plan to conduct three-five focus groups before and after the course, with four-six persons in each group. Each focus group will last 1.5-2 hours. The focus group interview will be loosely structured around an interview guide. The interview guide consists of both questions and ice-breaking exercises and focusing exercises [20].

The interviews will be led by investigating facilitator, observed and recorded in writing by an observer, and audio recorded and transcribed by a research assistant.

### Participant observation

Participant observation will be conducted on two of the MHFA courses, structured by an observation guide. The investigator will participate in the course on equal terms as the participants; participate in exercises and in discussions. This is to gain an understanding of the empirical setting in which data should be understood [20]. Further, participant observation will provide data on specific social negotiations of behavior and attitudes in the group of participants.

The notes will be transcribed shortly after and gathered in an early-analytical document in which observational notes and initial interpretations, analytic comments and views will be kept separately to avoid contamination of subsequent analysis.

### Analysis outline

Primary outcome in the quantitative study will be increased confidence in providing help in situations where people are in a mental health crisis or experiencing a mental health problem by comparing the intervention group after having attended the training course with the control group. Secondary outcome will be increase in knowledge about mental health problems and crisis and options for professional reference as well as an increase in positive attitudes towards people suffering from mental health problems also by comparison of the two groups. Power analysis was performed using PS – Power and Sample Size Calculation. We calculated that with alpha = 0.05, a power of 0.8 and a standard deviation 0.75, and a smallest clinically relevant difference in means between the groups of 0.19 we would require 250 persons per group.

Continuous outcome measures will be analyzed using generalized linear models including treatment allocation, test of interaction term between allocation and the vignette used, as well as variables associated with attrition or baseline imbalances between the groups, if any. Missing data is expected to be missing at random and handled using multiple imputations. Dichotomous outcome measures will be analyzed using binary logistic regression in the same manner. To describe the reliability of the data from the attitudes questions: “What you think” contra “what others think”, we will analyse by using Cronbach’s Alpha. The case and knowledge based items in the questionnaire will be ranked where high scores equal right answers. Data will be analysed using IBM SPSS version 22.

To take a systematic view on the qualitative data, we plan to use the software program Nvivo, which provide for a readily accessible categorizing and coding of the data. We will analyze the qualitative data using a phenomenological approach [17]. Here we will employ an emphasis on the structure of experience and consciousness centered on gaining knowledge and changing behavior. Based in phenomenology, the analysis will explore how knowledge and attitudes are produced and reproduced in ‘natural’ social situations. The analysis will thus be embedded in a perspective on the participants as a social group in an intersubjective life world. Thus, the analysis will both contain a characteristic of the participants as a social group, and an analysis of behavior and attitude. Lastly, the analysis of the qualitative data will present thoughts on contents of the educational programme.

Each set of data is independent, but provides a united understanding of the training program MHFA in a Danish context.

### Ethical considerations

According to Danish legislation approval from the ethics committee was not required. Notification and approval is only required when biological material involved. A training course, like the Mental Health First-Aid, is thought to have no side effects.

Data will not include detailed, sensitive personal, disease-related information. Participants in the focus groups will be asked to fill out a consent form. An advantage of focus groups as research method is that sensitive issues may be more readily discussed within these groups [17].

## Discussion

### Strengths and limitations

The randomization procedure offers strengths to this study by reducing the risk of selection bias. Equally, the analysis of the quantitative survey will be blinded with the intent of being unbiased.

The mixed-methods approach in the design of the study will allow us to qualify and further develop the results, and thereby present a more thorough evaluation of the MHFA training. The qualitative type of data gives greater depth, while the quantitative data gives greater breadth; together it yields results from which one can make better inferences [17].

The study may have some limitations. There can appear a risk of contamination between the intervention group and control group, since they will sometimes work at the same workplace. Previous studies have emphasized difficulties in obtaining adequate follow-up rates. However, in this study contacts are mainly on email, hopefully to prevent attrition bias, and missing data will be handled using appropriate statistical methods.

Furthermore, the study population represents a great part of people already working as ‘care takers’, which means that they may be especially motivated and already in line with providing help.

A limitation in the study can be that outcomes measured are sensitive to interpretation, hence a challenge to uniform. We hope that our use of a mixed-methods design in the study can strengthen the analysis and take up this challenge. In the evaluation study the quantitative research is suitable for examining the causal relationships between attending the training course and an increase in confidence and knowledge and a decrease in negative attitudes, whereas the qualitative research can provide a broader context for understanding of the data and come closer to the practice on the training course and behind the causal relationship found in the quantitative research [21].
